# Can we adapt fairly? Scoping review of health equity implications of flood risk in coastal communities

**DOI:** 10.1136/bmjph-2025-002588

**Published:** 2025-08-18

**Authors:** Grace Turner, Sari Kovats, Rachel Brisley, Sally Brown, Owen Landeg, Louise O’Connor

**Affiliations:** 1 Health Protection Research Unit in Environmental Change and Health, NIHR, London, UK; 2 Public Health, Environment and Society, London School of Hygiene & Tropical Medicine, London, UK; 3 Centre of Global Change and Health, London School of Hygiene & Tropical Medicine, London, UK; 4 Ipsos MORI UK Ltd, London, UK; 5 School of Geography and Environment, University of Southampton, Southampton, UK

**Keywords:** Public Health, Epidemiology, Emergencies, Scoping Review

## Abstract

**Background:**

As climate change progresses, it is critical to assess the equity of health impacts, adaptation interventions and policies. Climate change can contribute to coastal hazards like flooding resulting in loss of life, property and land, leading to potential long-term physical or mental health impacts. Additionally, some UK coastal populations often face social deprivation and limited healthcare access, which can be worsened by environmental changes.

**Methods:**

We conducted a scoping review of UK evidence on (a) inequalities in coastal flood risk and (b) the equity of measures to manage climate-related flood risks. Interventions included plans, flood insurance and infrastructure, including natural flood management. Following the screening of 19 329 references, we included 11 papers in the final review.

**Results:**

Four studies examined the differentials in current and future coastal flood impacts, and seven assessed the equity of adaptation measures. Coastal flood risk is unevenly distributed across the UK. Policies and practices like household insurance and property resilience measures may increase inequalities, while community engagement, planning and structural solutions can reduce disparities, depending on local context and implementation.

**Conclusions:**

Adaptation to UK coastal flood risk requires both short-term and long-term strategies. Approaches relying on individual behaviour or household income may worsen health inequalities. Further evaluations and better evidence are needed to improve flood planning and incident management. Climate change presents a challenge for organisations to deliver national and local policy responses ensuring that adaptation is effective and equitable in the immediate and longer term.

**WHAT IS ALREADY KNOWN ON THIS TOPIC**

- Coastal flooding undermines health and well-being. Many coastal areas with high levels of deprivation are also at high risk of flooding. In the UK, investment for coastal flood risk interventions including defences funded through government or partnership funding has been targeted at low-income, deprived communities, but evidence is less clear for other adaptation or resilience measures.

**WHAT THIS STUDY ADDS**

- A comprehensive scoping review of current evidence on inequalities in coastal flood risk and in adaptation responses for coastal communities in the UK. There is limited evidence on observed or modelled inequalities in health impacts from coastal flood hazards despite the high prevalence of social deprivation in coastal communities in the UK.

**HOW THIS STUDY MIGHT AFFECT RESEARCH, PRACTICE OR POLICY**

- Greater consideration of equity is needed in local and national coastal risk adaptation planning and intervention to address the exacerbation of health inequalities among coastal communities already experiencing challenges such as deprivation, ageing population and social decline. The evidence highlighted here can inform UK and other high-income country decision-making and policy implementation such as national and regional flood and coastal risk resilience plans, national climate change risk assessment frameworks and also flood and coastal risk mapping of the most vulnerable.

## Introduction

 Globally, coastal communities are increasingly at risk from flooding, which is being significantly exacerbated by climate change and sea level rise. One example is the UK coastline which is highly vulnerable to climate change, with coastal areas playing a vital role in the economy through agriculture, fishing, manufacturing, tourism, ports and energy generation. However, many historical UK seaside towns have experienced economic decline and social deprivation, leading to significant public health challenges.^1^,^4^ In addition to the lack of investment, remoteness, employment uncertainty and demographic changes, climate change is already affecting UK coastlines^5^ through sea level rise, storm surges, flooding (coastal, groundwater, surface water and fluvial), high winds and coastal erosion. As of January 2024, 2.6 million properties are in areas at risk of flooding from rivers and sea in England.^6^ Approximately 54 400 residential properties lie within England’s coastal floodplain, and 10% of these are at risk of flooding (1 in 75-year risk) with existing defences.^7^ Coastal climate change impacts will intensify in the future, creating an urgent need for effective and equitable adaptation measures to protect population health.^8^

Flooding causes mortality, injuries and mental health conditions^5^ as well as exacerbations of non-communicable diseases and increased contact with health services.^9^ Coastal flooding is often episodic, affecting the same communities repeatedly and, without warning or prior action being undertaken, can cause mass mortality. A global systematic literature review identified that flood events were associated with an increased prevalence of anxiety disorders in Spain, and post-traumatic stress disorder and depression in the USA and Australia.^10 11^ The review reported predictors of developing a mental health condition following a flood disaster include: precarious living conditions, low household income, displacement from home, persistent material damage and insurance-related issues.^9 10^ Coastal flooding is more dangerous than surface or fluvial flooding due to the depth and velocity of water.^12^ Managing flood risk involves national and local governments, emergency responders, healthcare, community groups and flood forums. In extreme cases, flooding can threaten the viability of coastal communities.^13^ Public investment currently prioritises deprived coastal areas for flood risk management solutions. Many urbanised coastal zones rely on traditional engineering to hold the coastline, as indicated in Shoreline Management Plans (SMPs) in England and Wales.^14^ A review of 19 EU coastal countries highlighted a progressive increase in the adoption of local adaptation plans and strategies addressing problems faced by coastal areas but a lack of attention to equity issues, such as preventing the exacerbation of pre-existing vulnerabilities in the most exposed coastal regions.^15^ Additionally, UK coastal flood defences are deteriorating due to ageing assets, limited maintenance funds and more extreme weather.^16^ New strategies are needed to enhance community resilience and address residual risks.

Social and health inequalities in the UK are widening. The Marmot Report on Inequalities 2020 advocates two key strategies: integrating equality and health equity into all policies (across all government departments) and implementing effective, evidence-based interventions and delivery systems.^17^ The distributional impacts of the evolving coastal flood risk on health in the UK need to be better understood^3^ and recently, there has been increased attention on the health risks faced by coastal communities in the UK.^3 4^ Public health and equity are not sufficiently considered in the implementation, efficacy and cobenefits of coastal adaptation strategies. At a minimum, these interventions should not disadvantage specific groups or worsen existing disadvantage. Governing bodies must balance adaptation with conflicting priorities, such as housing targets, economic challenges and limited funding. Additionally, local authorities’ capacity constraints have hindered recovery from coastal hazards.^1 8^ This paper aims to review the evidence on the differential health impacts of coastal flooding and assess the health equity implications of coastal flood adaptation responses in the UK.

## Methods

### Search strategy

A literature search was conducted in March 2023 to identify studies on the distributional health impacts of coastal flood hazards and health equity implications of coastal flooding adaptation. Database searches were limited to English-language studies from 2010 to 2023 inclusive. The search was limited to English-language studies from 2010 to 2023 due to a prior systematic review.^1^ Six databases were searched: PubMed, Global Health, Web of Science, Scopus, Social Policy & Practice and PsycINFO. Reference lists of included studies were handsearched, and authors were contacted for full texts if not openly available. The review followed the Preferred Reporting Items for Systematic Reviews and Meta-Analyses extension for Scoping Reviews (PRISMA) guidelines^18^ (online supplemental material S1). The search strategy, developed in PubMed, was adapted for other databases (online supplemental material S2).

### Selection criteria

Papers were managed using Endnote V.20.2.1 and Rayyan (https://rayyan.ai/). Following the reference search, all references to be screened were transferred to Endnote to be stored. These were then transferred to Rayyan (online screening tool) which allows for the user to select include or exclude when screening by title and abstract, then later by full text screening. All duplicates were removed, and titles and abstracts were dual-screened by GT and SK to minimise selection bias. Inclusion and exclusion criteria were followed rigorously during full-text screening by GT and LO’C, and SK was consulted as third reviewer to check for consistency and agree articles for the final review. Inclusion criteria were followed during article screening for the review (table 1). Using Rayyan, papers that were excluded during screening were assigned an exclusion reason using the labelling function, for example, not UK focused research. Relevant information extracted separately for the two research questions included: population, study characteristics, coastal hazard exposure, coastal adaptation response/measure, inequality dimension, data analysis methods, health outcome and study findings.

**Table 1:** Inclusion criteria for scoping review screening process of articles

Inclusion criteria	Description
Population	Residential and transient groups (eg, tourists, seasonal workers, second homeowners) in coastal communities
Setting	Coastal communities within UK (England, Northern Ireland, Scotland and Wales) Coastal community defined as ‘any coastal settlement within a local authority area whose boundaries include coastal foreshore, including local authorities whose boundaries only include estuarine foreshore. Coastal settlements include seaside towns, ports and other areas which have a clear connection to the coastal economy” as per the Coastal Communities Alliance.^49^ This was applied to all UK nations. UK, national, regional or community specific.
Exposure and risk	Flood risk, event or hazard (eg, coastal or fluvial flooding, storm surge, sea level rise, coastal erosion) on the coast.^50^ Implemented coastal flood risk management, response, policies, strategy or intervention. Coastal flood risk management aims to reduce the impacts of coastal flooding through adaptation measures including spatial planning, engineered hard and soft interventions, and insurance as well as utilising community resilience, for example, local flood forums. Incident management services provide warning of properties at risk from flooding within flood forecasting timescales.
Outcomes	Reported distribution of health outcome by inequality dimension: Direct health impact: being flooded, flood risk to health, death, injury, morbidity outcome (eg, hospital admission). Mental health outcome, including solastalgia defined as the distress that is produced by environmental change impacting on people while they are directly connected to their home environment.^51^ Indirect health impact, for example, health services disruption, population displacement, household income.^5^

We included only papers published in English, without limiting study design (eg, observational, modelling, intervention and qualitative studies). Flood risk is defined as a function of hazard, exposure and vulnerability. Areas at risk include current or future flooding from rivers, the sea, groundwater and surface water (pluvial). Health inequality refers to differences in health status due to experiences and opportunities faced by groups. We considered individual factors (protected characteristics), structural factors and socially excluded groups, such as people experiencing homelessness.^19^ Protected characteristics, covered by the Equality Act 2010 to prevent discrimination, include age, gender, race, disability, religion, beliefs and pregnancy. However, discrimination is only part of the broader causes of health and social inequalities. Structural inequalities manifest in access to housing, income, employment and services. Adaptation opportunities are unlikely to be equally distributed across the UK population. Coastal flood risk management and adaptation interventions were categorised into spatial planning, engineering, insurance and community resilience (table 2).

**Table 2:** Coastal adaptation approaches in England, adapted from van der Plank *et al*^52^

Spatial planning	Engineering	Insurance	Community resilience
The policy and practice of the organisation of the intended purposes for land, incorporating flood knowledge of areas to shape development plans and planned purposes for that space—to manage flood risk.	The use of soft and hard physical interventions, to support, maintain or develop existing natural or human risk-reducing features, applied to local to system scales—to manage flood risk.	Redistribution of the potential financial damages of flooding through the market. Can also be used to enable or discourage development in hazard areas, as well as to encourage property-level resilience—to manage flood risk. Reinsurance schemes to support transition to risk-reflective pricing of flood insurance	The ability of communities on a system level to withstand, adapt to, and recover from shocks (such as a flood event) in a way that it enables them to pursue their social, ecological and economic development objectives

### Analysis

We conducted a narrative synthesis of the findings due to variations in study design, methods, interventions and data sources. We separately analysed inequalities in the health impacts of coastal change and the equity implications of coastal adaptation. Outcome measures included health service use, flood risk, mental health, physical health, economic pain, financial loss and displacement. Key findings from the scoping review were mapped to dimensions of health inequality and flood risk interventions, with results reported by health outcome domain.

### Patient and public involvement

To inform the framing of the results in this review, a coastal and health equity discussion workshop was conducted with the Health Protection Research Unit on Environmental Change and Health public panel PLANET.^20^ The 25 public contributors provided useful insights and comments during the 1-hour online workshop and were reimbursed with a voucher of their choice for their valuable contribution to this research. The PLANET panel was sent a draft version of the manuscript to review.

## Results

**Figure 1:**
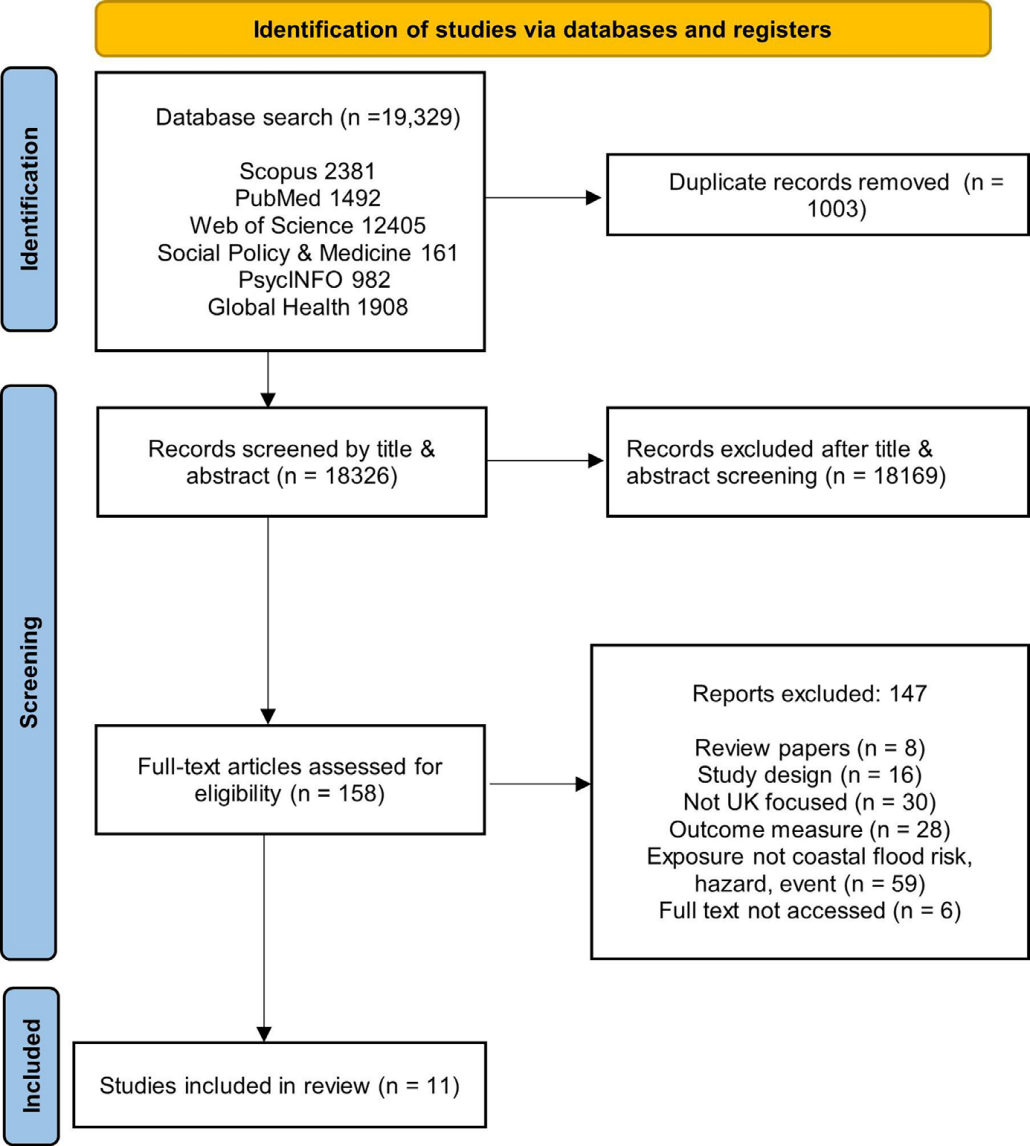
PRISMA diagram showing study number at each search stage. PRISMA, Preferred Reporting Items for Systematic Reviews and Meta-Analyses.

Following screening and the removal of duplicates, 11 articles were included in the final review (figure 1). Of the 147 excluded references, 59 did not analyse coastal flood risk, a hazard or event, 30 studies were not UK specific, 28 did not report on health inequalities as outcome, 16 were commentaries or overview papers, 8 were reviews and 6 were unavailable to access full text.

### Study characteristics and methods

All studies assessed either coastal or fluvial flooding in coastal areas. Three of the studies were focused on storms, whereas all others characterised flood risk by proximity to coast. No studies observed impacts from hazards other than coastal flooding, such as coastal erosion. We found four papers that quantified the distributional health impacts of flooding in coastal communities^21^,^24^ (table 3). Seven papers considered the health equity implications of coastal adaptation measures^25^,^31^ (table 4).

**Table 3:** Summary of studies that quantify distribution of coastal hazards on health inequalities

Author	Setting, time frame, sample size	Study design	Coastal hazard	Inequality measure	Analysis	Flood risk or health outcome
*Yu et al* ^21^	England Scenario-based study UK 2011 Census	Cross-sectional	Coastal flooding risk associated with annual Exceedance prob: >3.3% (high); between 3.3% and 1% (medium); between 1% and 0.1% (low); less than 0.1% (very Low)	Elderly, young children, people with bad health, households with deprivation, ethnicity	Network-based geospatial analysis	Ambulance response time to vulnerability hotspots (accessibility).
Garbutt *et al*^23^	Norfolk Scenario-based study 539 LSOAs	Cross-sectional	Tidal flooding	Vulnerability index—household composition, caregiving responsibilities, age, income, sex, education, disability, deprivation, car and food accessibility	Developed vulnerability score and used Norfolk as case study for testing indicator.	Flood risk and impact on travel time to hospital
Johnson and Yu^22^	Norfolk and Suffolk, UK Scenario-based study 550 care homes	Cross-sectional	Coastal and fluvial flooding (high > 1 in 30 years, medium 1 in 30 years—1 in 100 years and low 1–100 year—1 in 1000-year flood risk)	Vulnerable residents in care home	Network-based geospatial analysis	Coastal and fluvial flooding impact on the provision of ambulance-assisted evacuations; financial costs to National Health Service
Rözer and Surminski^24^	England and Wales 2008–2018 1.3 million built homes	Cross-sectional	Fluvial and coastal flooding	Resident groups: ‘Struggling’ homeowners, ageing manual labour, socioeconomic diversity, stable multicultural urban, upward thriving, stable affluent, rejuvenating	Analysis of flood exposure of newly built homes between 2008 and 2018 is distributed among various socioeconomic neighbourhood types.	Flood risk

LSOAs, lower-income neighbourhoods.

**Table 4:** Summary of studies that describe differential impact of coastal adaptation interventions

Author	Setting, time frame, sample size	Study design	Coastal hazard	Adaptation intervention	Inequality measure	Analysis	Flood risk or health outcome
Houston *et al*^29^	Scotland 1993–2005 593 flooded individuals	Cross-sectional	Coastal flooding	Insurance, flood warning system	Age, disability, car ownership, household income, occupation, housing tenure	Participant survey responses scored for ‘overall impact’. Multiple linear regression models.	Short term: financial loss, discomfort, displacement, stress Long term: deterioration in mental health and physical health, worry about future flooding
Kazmierczak *et al*^28^	Scotland Scenario-based study Scotland Census 2001 and 2011	Cross-sectional	Coastal flooding	Insurance	Social vulnerability index (age, health, housing, green space, income, information use, local knowledge, social networks, tenure, mobility, physical access, crime, access to services)	Social vulnerability to flooding index combined with flood hazard-exposure index under multiple return periods=index of flood disadvantage	Flood disadvantage index
Sayers *et al*^25^	UK Scenario-based study (2020s, 2050s, 2080s) UK population	Cross-sectional	Coastal flooding	Insurance	Social vulnerability, ethnicity, income	Future flood explorer modelling framework includes data on flood hazard exposure, vulnerability and adaptation strategies	Flood risk
Sayers *et al*^26^	UK Scenario-based study (2020s, 2050s, 2080s) UK population	Cross-sectional	Coastal flooding	River and coastal defences; spatial planning, building regs, forecasting and warning systems, insurance, community and emergency response	Neighbourhood Flood Vulnerability Index—age, health, income, information use, local knowledge, tenure, physical mobility, crime, housing characteristics, direct flood experience, service availability, social networks	Future flood explorer modelling framework includes data on flood hazard exposure, vulnerability and adaptation strategies	Social Flood Risk Index; Relative Economic Pain
Houston *et al*^30^	Scotland 2006 (1–12 years after flood) 1223 households	Cross-sectional	All flooding incl. coastal flooding	Property level protection, contents insurance, flood warning systems, emergency support during/post flood	Household composition, income, occupation, education, home ownership, house type, urban/rural	Multivariate logistic regression assessing flood measures.	Flood risk.
Landeg *et al*^27^	Boston, Lincolnshire During and after December 2013 flood 18 participants	Qualitative	Coastal flooding	Healthcare response, evacuation	Vulnerable individuals, hidden individuals (unregistered migrant workers)	Qualitative interviews and thematically analysed.	Healthcare system impact.
Penning-Rowsell and Pardoe^31^	UK 1983–2013 12 residential impact groups	Cross-sectional	Fluvial and coastal flooding	Flood risk management, insurance	Household income status, tenure	Scenario-based modelling changes of government investment to reduce flood risk and flood insurance	Flood risk

The studies in this review came from various populations: UK (n=3), Scotland (n=3), county (n=3), England (n=1) and England and Wales (n=1). The dimensions used to measure inequality varied significantly across studies. Seven studies disaggregated coastal flooding risk or impact by age, and studies also considered: household income (n=7), housing tenure (n=5), disability/mobility (n=6), those experiencing poor health (n=3), social vulnerability (n=3), household deprivation (n=2), ethnicity (n=2), car ownership (n=2), occupation (n=2), education (n=2), household type (n=2) and those with caregiving responsibilities (n=1). Four studies used composite vulnerability indices, for example, neighbourhood flood vulnerability index. Flood risk or health outcomes reported in the included studies were similarly heterogenous (tables3  4). The most common reported outcome was population flood risk as a proxy for health impact (n=7). Four of the 11 studies analysed health service delivery. Only one reported mental health impact, specifically stress, long-term deterioration of mental health and worry about future flooding as outcomes of interest.^29^ Three studies analysed the impact of flooding in coastal areas on a measure of financial impact: financial loss (n=1), costs to the National Health Service (n=1) and Expected Annual Damages/Relative Economic Pain (REP) (n=1). The adaptation interventions addressed included building or contents insurance/Flood Re (n=5), emergency planning response (n=3), flood warning systems (n=3), flood risk management (n=2), property level protection (n=1), evacuation (n=1), spatial planning and building regulations (n=1). 

### Narrative analysis

#### Equity implications of health impacts of flooding in coastal populations

Four studies assessed only flood risk to communities across the UK and England, finding that flood disadvantage was most acute at the coast (table 3).^22^,^2528^ National-level aggregate measures mask significant local disparities, for example, one-third of the population in coastal floodplains live in the 20% most deprived areas.^26 28^ Older adults (65+) and low-income groups in coastal communities were consistently at higher risk of adverse flooding impacts (n=6), with one study reporting deterioration in mental and physical health.^22^,^2426 28 29^ In Norfolk and Suffolk, 13% of care homes with older residents were at risk of fluvial and/or coastal flooding despite defences.^22^ In Scotland, coastal flooding caused higher disadvantage and social vulnerability in urban areas compared with rural areas.^28^

Differences in flooding impact by housing type or tenure are complex. From 2008 to 2018, 5% of 1.3 million homes in England and Wales were built in high-risk flood zones. New builds in high-risk flood zones, especially low-lying coastal areas in England, have increased. Development in flood-prone areas should be avoided, but if necessary, it must be ‘made safe for its lifetime without increasing flood risk elsewhere’.^24^ Homeownership was linked to greater short-term flood impacts, such as discomfort, stress and displacement, compared with rented accommodation in one Scottish study.^29^ A study on tidal flood risk in Norfolk found that lower-income neighbourhoods and those with more retired individuals were more likely to experience flooding than other areas.^23^

####  Equity implications of indirect health impacts in coastal regions

Overall, seven studies considered the distribution of indirect health impacts from flooding in coastal regions.^21^,^2325 26 29 31^ A reduction in ambulance service compliance with mandatory response times was observed across England during low-magnitude coastal and fluvial flooding.^21^ Older adults (65+) were disproportionately impacted by reduced emergency service coverage (including ambulance services).^21^ Two studies found that households or care home residents in rural or inaccessible areas faced significantly longer travel times to healthcare during all flood scenarios. One study identified that ethnic minorities and more deprived households were less affected by flood impacts on emergency service accessibility within 7 min and 15 min response times, likely due to living in urban areas.^21^

Five studies in this review observed differential economic impacts from coastal flooding.^22 25 26 29 31^ One study estimated that the sharpest rise in relative annual costs would affect financially deprived households, particularly those in public housing, under extreme scenarios (flood insurance and Flood Risk Management Plans fully based on flood risk), with costs over 50 times the baseline.^31^ The study concluded that a risk-reflective flood management approach could shift the burden onto financially deprived households, limiting their ability to access investment and insurance. Expected annual damages for coastal areas varied by flood magnitude and location, with rural areas typically facing higher costs.^26^ Social vulnerability categories showed limited variation in expected damages after a flood, but the most vulnerable coastal neighbourhoods likely faced twice the average economic pain.^25 26^ One study found increased NHS evacuation costs for care home residents, due to longer travel times as flood magnitude rose.^22^

####  Equity implications of coastal risk management

No studies directly addressed the impact of response measures on health. Six papers assessed the equity implications of coastal risk management or adaptation interventions (table 4).^25^,^31^ Most evidence focused on building and contents insurance. Five studies examined the health equity implications of flood insurance. One study found that over one-third of households in flood-prone areas lacked structure or contents insurance, and a shift to risk-based insurance could lead to higher costs.^25^ Having contents insurance was positively linked to higher income, homeownership or prior flooding^30^; however, black or other ethnic minority groups^25^ are less likely to have flood insurance and experienced higher levels of disadvantage.^28 29^ Lower-income groups with limited contents insurance in socially vulnerable areas experienced a higher relative impact from flooding in one study^26^ but showed no association in another.^29^

There is limited evidence of inequalities in access to flood warnings. Demographic, social and housing tenure differences were not predictors of receiving a flood warning or assistance.^30^ However, households with a disabled person were less likely to report receiving assistance from emergency responders.^30^ The survey, not limited to coastal flooding, showed less than 50% uptake of property flood resilience (PFR) measures among flooded households. Note that PFR is not considered a key intervention for coastal flooding. Those with children, a mortgage or in terraced housing or flats were less likely to implement flood alleviation measures.^30^ The paper highlights complexities in social housing and surface water flood risk management, finding council tenants more likely to have flood alleviation measures postflood, but less likely before a flood, which indicated that local governments act to implement property-level flood alleviation measures after a flood but not before a flood. One study noted challenges in identifying flood vulnerability within communities, such as ‘hidden populations’, leading to miscommunication between responders.^27^ Other barriers to evacuation included overcrowded housing, refusal to evacuate, non-English language, limited mental capacity and complex healthcare needs.

A full summary of the findings is provided in online supplemental material S3.

## Discussion

Despite the importance of equity in adaptation, we found limited evidence on the health impacts of flooding or the equity implications of coastal risk management in the UK. This includes the full hazard to impact chain, and the full understanding of the variables that could improve or exacerbate impacts that could directly influence short-term and long-term health outcomes (eg, duration of disturbance, removal of damp from homes, loss of income, access to mental and other health services). Although the evidence is limited, we found that UK coastal communities face increased disadvantages from coastal flooding, with the most socially vulnerable neighbourhoods at disproportionately higher risk, consistent with existing literature.^1 2 5 13^ This disparity arises from increased exposure, extreme weather and variable access to risk management and resilience measures. Many UK coastal regions already face economic and social decline, so climate-driven flood risks may worsen these disadvantages.^5^ A large body of evidence exists on inequalities related to inland flooding which are different from coastal flooding in the UK context.^32^ Evidence from global literature highlights poorer residents and older adults have a higher risk during and after coastal and river flood events in the USA as well as consequences for communities in disruption to healthcare delivery and infrastructure following flood events.^9^ Like this review, flood impacts vary by flood type, but the most affluent are typically more exposed to river flooding.^33^ This review highlights an important gap in the understanding of the distributional health impacts of coastal flooding in the UK.

This review found that socioeconomic and demographic factors influenced the health impacts of coastal flooding. Age, income, caregiving responsibilities, limited household amenities and distance from services were linked to short-term and long-term impacts, such as financial loss, mental and physical health decline and displacement. Similar findings were seen in studies on psychosocial symptoms from all flood sources in England.^34^ The proposed decommissioning of Fairbourne, Wales, by mid-century showed high levels of stress and mental health decline among residents, sparking debate and exploration of innovative adaptation approaches.^35^ Homeownership was linked with greater short-term flood impacts, but financially secure homeowners recovered more quickly, likely depending on insurance coverage.^35^ Feedback from Fairbourne residents highlighted increased stress and anxiety due to limited capacity to address issues raised in the SMP.^36^ Some coastal communities in other countries report solastalgia, distress from environmental change, though it is less common in the UK depending on flood events.^37^,^39^ Furthermore, social, health and welfare providers’ engagement is crucial in developing decision pathways for Fairbourne and similar at-risk communities. This review found reduced spatial coverage and increased travel times for emergency services during coastal flooding. Inaccessible or remote coastal communities, including care homes, were most affected. Older adults, especially in rural areas or coastal care homes, were disproportionately impacted by reduced emergency service coverage, while ethnic minorities and deprived households in urban areas were less likely to experience service inaccessibility. Emergency planning should consider service accessibility to vulnerable groups during floods.

Financial costs from coastal flooding are expected to be higher for deprived households, with the most socially vulnerable neighbourhoods facing twice the average REP. The economic impacts may stem from limited insurance coverage, as around 32% of households in flood-prone areas are uninsured.^7 26^ This review shows that contents insurance uptake is higher among high-income households, homeowners or those previously flooded, while black and other ethnic minorities are less likely to have insurance and face greater flood disadvantage. Lower-income groups with low contents insurance uptake experience higher relative flood impacts in vulnerable neighbourhoods. Previous research supports this, showing differential insurance uptake by income. Across Europe, evidence indicates regional inequalities in the ability to use flood insurance as a mechanism for flood risk adaptation, showing an unaffordability and declining demand for flood insurance towards 2080.^40^ The proposed cessation of Flood Re in 2039 may worsen the lack of flood insurance uptake among vulnerable groups already at risk.^41 42^ Housing type and tenure were linked to higher property-level flood alleviation rates, though this varied for council tenants depending on previous flood experience.

This review highlights significant gaps in our understanding of flood impacts on health, and how these change with time since the occurrence of the flood. Further evidence is needed on UK health-related impacts, measures to improve impacts and the equity implications of these, most notably planning and preparedness including the role of community resilience. Existing literature has examined community flood groups in inland communities in Yorkshire, UK^43^ and elsewhere in the UK^44^ indicating that demographic diversity and representation can have variable effects on flood risk management and resilience to embrace wider social justice issues. The studies highlighted that bottom-up initiatives, including inclusion of coastal groups, have been a successful and complementary mechanism to support top-down governance frameworks and flood-risk management schemes for inland flooding: while they may not be able to change a policy, they have helped to create a better environment to engage and participate in the process of change. Considering the recognised disparities and shifting demography of coastal communities, initiatives like community flood groups can be an effective approach for coastal flood risk management. However, it is clear this is dependent on social capital, representativeness, differential risk in health impacts and wider vulnerabilities. Some countries are developing tools to identify population groups experiencing negative flood impacts to inform disaster risk management.^45 46^ Furthermore, the implications of engineering solutions such as hard coastal defences have traditionally led communities to believe they are ‘safe’ from a flood, with scepticism over nature-based solutions. However, recent evidence suggests growing appreciation of natural defences, for long-term thermal comfort, pollution and mental health,^38^ but also when integrated with hard structures, such as in tsunami-prone regions in Japan.^47^

This scoping review followed the PRISMA guidelines and included six databases from various research disciplines. To our knowledge, this is the first review analysing the equity implications of coastal change impacts and health risks, as well as the distributional impacts of coastal risk management and adaptation strategies on health in the UK. Due to limited data on health impacts from flood exposure, we used flood risk as a proxy measure for health impact. The findings from this review can inform future government and local authority adaptation strategies, ensuring all groups are considered and identifying strategies that may create inequalities. Some studies in this review modelled the distributional health impacts of coastal change through spatial mapping, but they focused on flood risk exposure rather than specific flood events. Flood risk mapping has methodological limits, as it classifies all populations living in flood zones as ‘at-risk.’ Future research could quantify distributional health impacts by analysing specific flood events and associated physical and mental health outcomes.

## Conclusions

Overall, this paper highlights the limited evidence of spatial and temporal distributional differences in coastal flood risk and health impacts among UK coastal communities. Adaptation to coastal flood risk currently involves a range of short-term and long-term approaches that may either exacerbate or address current inequalities. Adaptation strategies relying on individual behaviour change, insurance or retrofitting could worsen inequalities within coastal communities. Providing more in-depth impact evaluations of coastal flood risk interventions and management on health equity outcomes is needed to support flood planning, particularly in areas that may be facing deprivation and social decline. Further assessment of large-scale coastal flood risk resilience projects and equitable, accessible financial protection is crucial for understanding health outcomes and addressing coastal inequities.^48^ Climate change presents a challenge for organisations to implement national and local policies ensuring effective and equitable adaptation.

## Supplementary material

10.1136/bmjph-2025-002588 online supplemental file 1

## Data Availability

Data sharing not applicable as no datasets generated and/or analysed for this study.
