# Supplementary material for: Can we adapt fairly? Scoping review of health equity implications of flood risk in coastal communities
Source: BMJ Public Health. 2025 Aug 18;3(2):e002588. doi: 10.1136/bmjph-2025-002588 (PMC12366612; doi:10.1136/bmjph-2025-002588)
Supplement: online supplemental file 1 [file bmjph-3-2-s001.docx]

**Supplementary material - Can we adapt fairly? Scoping review of health equity implications of flood risk in coastal communities**

**S1 – PRISMA guidelines**

**Preferred Reporting Items for Systematic reviews and Meta-Analyses extension for Scoping Reviews (PRISMA-ScR) Checklist**

| **SECTION** | **ITEM** | **PRISMA-ScR CHECKLIST ITEM** | **REPORTED ON PAGE #** |
| --- | --- | --- | --- |
| **TITLE** | | | |
| Title | 1 | Identify the report as a scoping review. | 1 |
| **ABSTRACT** | | | |
| Structured summary | 2 | Provide a structured summary that includes (as applicable): background, objectives, eligibility criteria, sources of evidence, charting methods, results, and conclusions that relate to the review questions and objectives. | 2 |
| **INTRODUCTION** | | | |
| Rationale | 3 | Describe the rationale for the review in the context of what is already known. Explain why the review questions/objectives lend themselves to a scoping review approach. | 5 |
| Objectives | 4 | Provide an explicit statement of the questions and objectives being addressed with reference to their key elements (e.g., population or participants, concepts, and context) or other relevant key elements used to conceptualize the review questions and/or objectives. | 5 |
| **METHODS** | | | |
| Protocol and registration | 5 | Indicate whether a review protocol exists; state if and where it can be accessed (e.g., a Web address); and if available, provide registration information, including the registration number. | n/a |
| Eligibility criteria | 6 | Specify characteristics of the sources of evidence used as eligibility criteria (e.g., years considered, language, and publication status), and provide a rationale. | 6 |
| Information sources* | 7 | Describe all information sources in the search (e.g., databases with dates of coverage and contact with authors to identify additional sources), as well as the date the most recent search was executed. | 5-6 |
| Search | 8 | Present the full electronic search strategy for at least 1 database, including any limits used, such that it could be repeated. | S2 |
| Selection of sources of evidence† | 9 | State the process for selecting sources of evidence (i.e., screening and eligibility) included in the scoping review. | 6, S2 |
| Data charting process‡ | 10 | Describe the methods of charting data from the included sources of evidence (e.g., calibrated forms or forms that have been tested by the team before their use, and whether data charting was done independently or in duplicate) and any processes for obtaining and confirming data from investigators. | 7 |
| Data items | 11 | List and define all variables for which data were sought and any assumptions and simplifications made. | n/a |
| Critical appraisal of individual sources of evidence§ | 12 | If done, provide a rationale for conducting a critical appraisal of included sources of evidence; describe the methods used and how this information was used in any data synthesis (if appropriate). | n/a |
| Synthesis of results | 13 | Describe the methods of handling and summarizing the data that were charted. | 7 |
| **RESULTS** | | | |
| Selection of sources of evidence | 14 | Give numbers of sources of evidence screened, assessed for eligibility, and included in the review, with reasons for exclusions at each stage, ideally using a flow diagram. | 8 |
| Characteristics of sources of evidence | 15 | For each source of evidence, present characteristics for which data were charted and provide the citations. | 9 |
| Critical appraisal within sources of evidence | 16 | If done, present data on critical appraisal of included sources of evidence (see item 12). | n/a |
| Results of individual sources of evidence | 17 | For each included source of evidence, present the relevant data that were charted that relate to the review questions and objectives. | 13-15 |
| Synthesis of results | 18 | Summarize and/or present the charting results as they relate to the review questions and objectives. | 13-15 |
| **DISCUSSION** | | | |
| Summary of evidence | 19 | Summarize the main results (including an overview of concepts, themes, and types of evidence available), link to the review questions and objectives, and consider the relevance to key groups. | 15-17 |
| Limitations | 20 | Discuss the limitations of the scoping review process. | 17 |
| Conclusions | 21 | Provide a general interpretation of the results with respect to the review questions and objectives, as well as potential implications and/or next steps. | 18 |
| **FUNDING** | | | |
| Funding | 22 | Describe sources of funding for the included sources of evidence, as well as sources of funding for the scoping review. Describe the role of the funders of the scoping review. | 18-19 |

**S2 – Full search strategy for scoping review**

**Aim: review the evidence on the impact of climate change and implemented adaptative strategies on health inequalities in coastal communities the UK**

Databases:

- Pubmed
- Global health
- Web of science
- Scopus
- Social policy & practice
- PsychINFO

***Inclusion and Exclusion Criteria***

Publications qualified against the inclusion criteria if peer-reviewed. Limits were set for study design; observational quantitative studies and mapping or modelling studies were included if they used empirical data for a specific location. Commentaries, qualitative, opinion pieces, reviews, and editorials were excluded.

| **Inclusion criteria** | **Description** |
| --- | --- |
| Population | Residential and transient groups (e.g. tourists, seasonal workers, second home owners) in coastal communities |
| Setting | - Coastal communities within United Kingdom (England, Northern Ireland, Scotland, Wales) - Coastal community defined as “any coastal settlement within a local authority area whose boundaries include coastal foreshore, including local authorities whose boundaries only include estuarine foreshore. Coastal settlements include seaside towns, ports and other areas which have a clear connection to the coastal economy” as per the Coastal Communities Alliance. This was applied to all UK nations. - UK, national, regional or community-specific. |
| Exposure | 1. Flood risk, event or hazard (e.g. coastal or fluvial flooding, storm surge, sea level rise, coastal erosion) on the coast 2. Implemented coastal flood risk management, response, policies, strategy or intervention. Coastal flood risk management aims to reduce the impacts of coastal flooding through adaptation measures including spatial planning, engineered hard and soft interventions, and insurance as well as utilising community resilience e.g. local flood forums. Incident management services provide warning of properties at risk from flooding within flood forecasting timescales. |
| Outcomes | Reported distribution of health outcome by inequality dimension:   - Direct health impact: being flooded, flood risk to health, death, injury, morbidity outcome (e.g. hospital admission), - Mental health outcome, including Solastalgia defined as the distress that is produced by environmental change impacting on people while they are directly connected to their home environment­ - Indirect health impact: health services disruption, population displacement, household income. |

***Search Terms***

| *Coastal change (flood hazard)* | | *Environmental Health inequality* | |
| --- | --- | --- | --- |
| Coast*  Coastal climate change  Coastal change  Coastal risk  Coastal hazard  Environmental risks  Coastal movement  Seaside  Seaside decline  Flood*  Coastal flood*  Runoff beach  Coastal erosion  Shoreline erosion  Beach erosion  Sea level rise  Rising sea level  Sea level change  Coastal displacement | Storm  Storm surge  Ocean acidification.  Marine eutrophication  Sea temperature  Temperature rise  Temperature change  Wind  Precipitation  Heavy rainfall  Marine change  Wave  Wave size  Water salinity  Salinisation  Coastal morphology  Sediment supply | Coastal ineq*  Climate ineq*  Health ineq*  Climate change ineq*  Coastal vulnerab*  Environmental health inequality  Environmental justice  Climate justice  Coastal justice  Inequality  Inequity  Equity  Health disparities  Socioeconomic  Coastal commun*  Coastal group  Disadvantaged coastal commun*  Disadvantaged commun* | Sociodemographic  Geographical disadvantage  Relocation  Environmental poverty  Low income  Deprivation  Decline  Vulnerab*  Distributional differences  Distributional health impacts  Health differences |

**PubMed (example search)**

(((coast* OR "coastal climate change"[Title/Abstract] OR "coastal change"[Title/Abstract] OR "coastal risk"[Title/Abstract] OR "coastal hazard"[Title/Abstract] OR "environmental risks"[Title/Abstract] OR "coastal movement"[Title/Abstract] OR "seaside"[Title/Abstract] OR "seaside decline"[Title/Abstract] OR flood* OR "coastal flood*"[Title/Abstract] OR "runoff"[Title/Abstract] OR "beach erosion"[Title/Abstract] OR "coastal erosion"[Title/Abstract] OR "shoreline erosion"[Title/Abstract] OR "sea level rise"[Title/Abstract] OR "rising sea level"[Title/Abstract] OR "sea level change"[Title/Abstract] OR "coastal displacement"[Title/Abstract] OR storm OR "storm surge"[Title/Abstract] OR "ocean acidification"[Title/Abstract] OR "marine eutrophication"[Title/Abstract] OR "sea temperature"[Title/Abstract] OR "temperature rise"[Title/Abstract] OR "temperature change"[Title/Abstract] OR wind OR precipitation OR "heavy rainfall"[Title/Abstract] OR "marine change"[Title/Abstract] OR wave OR "wave size"[Title/Abstract] OR "water salinity"[Title/Abstract] OR salinisation OR "coastal morphology"[Title/Abstract] OR "sediment supply"[Title/Abstract]))) AND (("coastal ineq*"[Title/Abstract] OR "climate ineq*"[Title/Abstract] OR "health ineq*"[Title/Abstract] OR "climate change ineq*"[Title/Abstract] OR "coastal vulnerab*"[Title/Abstract] OR "environmental health inequality"[Title/Abstract] OR "environmental justice"[Title/Abstract] OR "climate justice"[Title/Abstract] OR "coastal justice"[Title/Abstract] OR "health disparities"[Title/Abstract] OR "coastal commun*"[Title/Abstract] OR "coastal group"[Title/Abstract] OR "disadvantaged coastal commun*"[Title/Abstract] OR "disadvantaged commun*"[Title/Abstract] OR sociodemographic OR "geographical disadvantage"[Title/Abstract] OR relocation OR "low income"[Title/Abstract] OR deprivation OR decline OR vulnerab* OR "distributional differences"[Title/Abstract] OR "distributional health impacts"[Title/Abstract] OR "health differences"[Title/Abstract]))) AND ((UK[Title/Abstract] OR england[Title/Abstract] OR english[Title/Abstract] OR wales[Title/Abstract] OR welsh[Title/Abstract] OR scotland[Title/Abstract] OR scottish[Title/Abstract] OR northern[Title/Abstract] OR ireland[Title/Abstract] AND northern[Title/Abstract] OR irish[Title/Abstract] OR "united kingdom"[Title/Abstract] OR "great britain")[Title/Abstract])

**S3 – Summary of key finding from studies**

| **Domain** | **Results summary** |
| --- | --- |
| Flood risk | - Flood disadvantage across all flood sources, most acute at the coast – 1.8 million people live in coastal flood plain - Those living in coastal areas, including residents in care homes, are at an increased disadvantage to coastal flooding in Scotland and England - Expected increase in new build developments in high risk flood zones, especially in lower lying costal areas in England |
| Inequality dimensions of flood risk/impact | - Social vulnerability to flooding and flood disadvantage has a strong coastal dimension – 33% of population living in coastal flood plain are within the 20% most vulnerable neighbourhoods - Disproportionate flood risks faced by socially vulnerable neighbourhoods in coastal areas is predicted to increase in the future - Age (elderly population), income, living alone, resident in care home in coastal area, lack of central heating, in poor health, being a carer, living far away from key services and depth of flooding were linked with short- or long-term flood impacts - Limited mobility being associated with coastal flood impacts varied across papers. - Having children and car ownership were not linked with short- or long-term impacts - Owning a home has been linked with greater short-term impacts from flooding. - Disproportionately lower share of new homes in high flood risk zone in England and Wales built in areas with increasingly struggling home-owners but also those classified as upward thriving neighbourhood types - Disproportionately higher share of new homes built in high risk flood zones in ageing manual labour and stable affluent neighbourhood types. - Hidden populations e.g. migrant workers and those with English as second language challenged communication of flood risk |

Table 1a: Summary of result from review on direct impacts from coastal change (flood risk) and equity dimensions.

| **Domain** | **Results summary** |
| --- | --- |
| Healthcare service delivery impacts | - Coastal flooding and fluvial flooding in coastal areas may have significantly reduced spatial coverage or no access to emergency services in all population groups. - More care homes became inaccessible during floods of all magnitudes and within all-response time targets – inaccessible care homes predominantly in coastal locations - Elderly population disproportionately impacted by reduced emergency service coverage (including ambulance service) in coastal areas during flood - Increases in travel time to hospitals in both rural and urban areas during flooded scenarios, rural areas experienced higher percentage increase - Differences by ethnicity in ambulance service accessibility and response times during flood. - More deprived households were less impacted by reductions in emergency accessibility during flood |
| Economic impact from coastal change | - Sharp rise in predicted annual costs for financially deprived households, during and following floods - The Expected Annual Damages for those living in coastal flood risk areas is ranged from £21 to £76 per person across papers in the review - Little fluctuation between social vulnerability categories in expected annual damages - However, in areas prone to coastal/tidal flooding, the most socially vulnerable neighbourhoods likely experience twice the average Relative Economic Pain - Increase in NHS costs for evacuations as flood magnitude increases due to increased vehicular travel time. |

Table 1b: Summary of results from review on indirect impacts from coastal change (flood risk) and equity dimensions.

| **Domain** | **Results summary** |
| --- | --- |
| Overall flood management and response | - Half of previously flooded households have undertaken property-level flood alleviation measures - 32% of households living in areas exposed to significant fluvial or coastal flooding may be without insurance - Preparedness, recognition of flood risk and awareness of potential impacts of the surge were inconsistent across health care system. - Significant challenges identified in defining flood vulnerability leading to miscommunication between frontline responders and those coordinating joint response to flooding – following floods GPs developed vulnerability criteria for future flood management - Challenges in emergency response due to densely occupied housing, refusal to evacuate and complex health care as well as routine health care disruption due to evacuation - Risk-based approaches to flood-risk management may lead to rises in costs per household |
| Inequality dimensions of coastal management and response | - Homeowners without a mortgage, council tenants and residents in detached or semi-detached homes displayed higher rates of property-level flood alleviation - Council tenants living in properties with property-level flood alleviation differed from pre-flood to post-flood - Prevalence of contents insurance is positively associated with higher income, homeownership, having previously been flooded but Black or from other Minority Ethnic groups are less likely to have flood insurance and have higher levels of disadvantage - Lower income and low contents insurance penetration lead to relative impact of a flood being higher in socially vulnerable neighbourhoods - No demographic, social or housing tenure differences in the likelihood of reporting receiving a flood warning or receiving assistance - Some evidence of households with a disabled person being less likely to report having received assistance in a flood - Those with limited mental capacity and the right to palliative patients to refuse evacuation challenged evacuation process |

Table 1c: Summary of results from review on flood risk management and response in coastal areas and inequality dimensions
